# Case Report: Mycosis fungoides as an exclusive manifestation of common variable immunodeficiency in a family with a *NFKB2* gene mutation

**DOI:** 10.3389/fonc.2023.1248964

**Published:** 2023-09-13

**Authors:** María Noel Spangenberg, Sofía Grille, Camila Simoes, Mariana Brandes, Joaquín Garcia-Luna, Ana Inés Catalán, Sabrina Ranero, Matilde Boada, Andreína Brugnini, Natalia Trias, Daniela Lens, Víctor Raggio, Lucía Spangenberg

**Affiliations:** ^1^ Cátedra de Hematología, Hospital de Clínicas, Facultad de Medicina, Universidad de la República, Montevideo, Uruguay; ^2^ Departamento Básico de Medicina, Hospital de Clínicas, Facultad de Medicina, Universidad de la República, Montevideo, Uruguay; ^3^ Bioinformatics Unit, Institut Pasteur de Montevideo, Montevideo, Uruguay; ^4^ Departamento de Genética, Facultad de Medicina, Universidad de la República, Montevideo, Uruguay

**Keywords:** human genomics, hereditary hematological disorders, mycosis fungoides, CVID, bioinformactics

## Abstract

**Background:**

Common variable immunodeficiency disorders (CVIDs), which are primary immunodeficiencies characterized by the failure of primary antibody production, typically present with recurrent bacterial infections, decreased antibody levels, autoimmune features, and rare atypical manifestations that can complicate diagnosis and management. Although most cases are sporadic, approximately 10% of the patients may have a family history of immunodeficiency. Genetic causes involving genes related to B-cell development and survival have been identified in only a small percentage of cases.

**Case presentation:**

We present the case of a family with two brothers who presented with mycosis fungoides as an exclusive symptom of a common variable immunodeficiency disorder (CVID). Whole-exome sequencing of the index patient revealed a pathogenic variant of the *NFKB2* gene. Based on this diagnosis and re-evaluation of other family members, the father and brother were diagnosed with this rare immune and preneoplastic syndrome. All CVID-affected family members presented with mycosis fungoides as their only symptom, which is, to the best of our knowledge, the first case to be reported.

**Conclusion:**

This case highlights the importance of high-throughput sequencing techniques for the proper diagnosis and treatment of hereditary hematological disorders.

## 1 Introduction

Common variable immunodeficiency disorders (CVIDs) are a group of primary immunodeficiencies associated with impaired B-cell differentiation and defective primary antibody production (1). CVID is characterized by heterogeneous clinical manifestations including recurrent infections, chronic lung disease, autoimmune disorders, gastrointestinal diseases, and heightened susceptibility to lymphoma (2). CVID is usually sporadic; patients usually have no reported family history of immunodeficiency. Nevertheless, approximately 10% of the patients may have other first-degree relatives with hypogammaglobulinemia or selective IgA deficiency (3). Monogenic causes of the disease, showing an autosomal dominant inheritance pattern, have been identified in less than 10–15% of all CVID cases and include mutations in genes involved in lymphoid organogenesis, B-cell survival, and maturation (4). In most cases, the causative mutations remain unknown, making it difficult to elucidate the pathogenic mechanisms of CVID (5). Next-generation sequencing (NGS) has revealed several genes associated with the pathology (6), including *TACI, CD19, BAFFR, CD20, CD81, CD21, LRBA, NFKB2, IL21, NFKB1, IKZF1, IRF2BP2*, among others. Several nonsense (7, 8) and less frequent missense mutations (9) have been reported as potential causes of CVID. Heterozygous *NFKB1* and *NFKB2* variants, collectively account for the most frequent genetic cause in CVID (9).

In particular, *NFKB2* is a widely studied transcription factor with key roles in the functioning of the immune system (2, 10), such as regulation of pathways leading to inflammation, cell survival, and differentiation (10). NF-κB2 is the principal protein involved in the noncanonical NF-κB pathway, is evolutionarily conserved, and functions in peripheral lymphoid organ development, B cell development, and antibody production (2). Mutations in the *NFKB2* gene can lead to a deficiency in NFKB2 protein, which is associated with CVID (2, 11). The exact mechanisms by which *NFKB2* mutations cause CVID are not fully elucidated, but it is thought that *NFKB2* plays a role in the development and function of B cells and T cells (12), which may lead to a deficiency in antibodies (13). Efforts have been made to formulate diagnostic criteria to better define this heterogeneous group of pathologic entities, including the ESID/Pan American Group of Immune Deficiency (PAGID, 1999) criteria (14), Ameratunga and colleagues (2013) criteria (15), and the most recent CVID International Consensus (ICON, 2016) criteria (16). Currently, all definitions of CVID exclude patients with known disorders; therefore, if a specific genetic variant is found (*NFKB1, NFKB2* etc.), the patient would be excluded from a CVID diagnosis and would then be reclassified as having a CVID-like disorder caused by a specific PID (primary immunodeficiency disorder)/inborn error of immunity (17).

CVID can be divided into two groups according to clinical manifestations. The most frequent group (more than 70% of patients) exclusively presents with infections, whereas the second, less frequent group has a variety of noninfectious inflammatory, autoimmune, and/or lymphoproliferative presentations, which are also associated with systemic immune activation (18, 19). In some cases (approximately 25%), patients in the second group may have inflammatory symptoms as the only symptom, with no obvious susceptibility to infectious disease. Patients with inflammatory/autoimmune features have increased morbidity and mortality; hence, several studies have focused on these groups to understand its heterogeneity (20).

Mycosis fungoides (MF) is a primary cutaneous T-cell lymphoma associated with the clonal proliferation of epidermotropic T-lymphocytes residing in the skin. MF accounts for more than half of all cutaneous lymphomas (21), and its reported frequency varies worldwide (22). The prevalence in the United States is ~0.58 cases per 100,000 person/years (23), and across Europe varies between 0.2-0.38 per 100,000 persons/year (24, 25), while in Uruguay, the prevalence is not well documented. In some cases, MF transforms into a more aggressive lymphoma that requires systemic chemotherapy, and the frequency of such transformations has been reported to range from 8% to 55% (26–29).

Here, we present the case of a family with two brothers who presented with mycosis fungoides as an exclusive symptom of CVID. Diagnosis was made via exome sequencing which revealed pathogenic variants in the *NFKB2* gene. Based on this diagnosis and re-evaluation of other family members, the father and brother were also diagnosed with this rare immune and neoplastic syndrome. All CVID-affected family members presented with mycosis fungoides as their only symptom, which is, to the best of our knowledge, the first case series to be reported.

## 2 Case series

### 2.1 Index case

**Figure 1:**
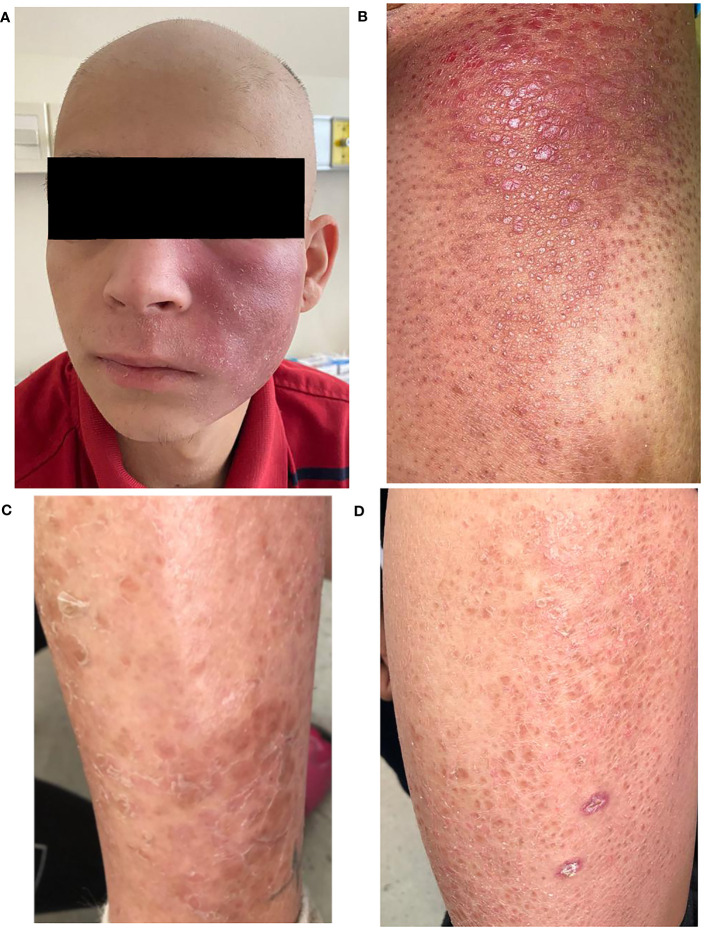
Clinical presentation of the patient. **(A)** Folliculotropic mycosis fungoides with lesions in the cheek. **(B)** Tumoral lesion of the limb after first line treatment. **(C)** Left lower leg skin lesion of brother (patient 2). **(D)** Left thigh skin lesion of brother (patient 2).

A 20 year-old, male patient has been referred to our hematological service from the dermatological unit with a diagnosis of folliculotropic mycosis fungoides. He has no comorbidities and no history of infections or autoimmune diseases. He has had a long history of generalized skin lesions and erythema since the age of nine treated with topical corticosteroids. Since 2 years ago, he began to notice the appearence of new lesions in different sites. At the time of referral to our service, he presented with generalized hairless skin, erythematous lesions in the armpit, in the left cheek and in the right thigh (**Figures 1A, B**).

The lesion on the right thigh became tumorous with progressive growth of;approximately 20 X 25 cm hence, aggressive transformation was ruled out upon referral to our department.

Right thigh biopsy suggested a mature non-Hodgkin lymphoma-T-helper phenotype (CD3+, CD4+, CD5+, CD7+, and CD8-), and negative for CD30. Thus, the diagnosis of mycosis fungoides with large cell transformation was confirmed.

Peripheral blood study shows no Sézary cells by cytomorphology or flow cytometry.

The PET scan showed the area of uptake in the above mentioned skin lesions (SUV between 2 to 3.5), hypermetabolic consolidation in the lower pulmonary lobe (SUV 2.5), and pleural thickness with an SUV of 2.7.

In conclusion, mycosis fungoides with large-cell transformation was verified with visceral involvement (pulmonary) without Sézary syndrome.

Treatment included local dermatological treatment and two initial cycles of chemotherapy with cyclophosphamide, doxorubicin, etoposide, vincristine, and prednisone (CHOEP), after one and a half month, we changed the therapeutic approach to gemcitabine monotherapy.

Clinical progression during treatment, with enlargement of the skin lesions and known pulmonary consolidation, was confirmed. Third-line treatment was performed based on photobeam and subsequently pembrolizumab (2 mg/kg IV) every 3 weeks. Now, one year later, he is receiving the 5th cycle of pembrolizumab with a partial response, and we plan to perform haploidentical allogeneic stem cell transplantation with his mother.

### 2.2 Patient 2

The brother had a history of few cutaneous lesions over the years, mostly in both legs, erythematous, not pruriginous (he does not remember the exact timeline of events), he never consulted because of them (**Figures 1C, D**). He has no prior history of infections or autoimmune diseases. After his brother’s diagnosis, he was referred for consultation to the dermatology service, and a cutaneous biopsy was performed and the diagnosis of folliculotropic mycosis fungoides was made.

A hereditary disorder became evident, but a specific diagnosis seemed impossible given the huge genetic heterogeneity and phenotypic overlap of these disorders; hence, we performed whole-exome sequencing (WES) on both siblings to obtain a specific molecular diagnosis.

### 2.3 Patient 3

The father has a history of cutaneous lesions, with the same features as described in the second son. He did not want any follow up but he agreed to a cutaneous biopsy of one of the lesions. The diagnosis of mycosis fungoides also was made. He dropped out of follow up.

## 3 Results

### 3.1 Whole exome sequencing revealed a heterozygous mutation in *NFKB2* gene in both siblings

We performed exome sequencing as part of a local pilot program aimed at promoting the use of high-throughput sequencing for the diagnosis of rare genetic diseases, with a special focus on hematological diseases. Informed consent to participate in the study and its publication was obtained from the participants and their families. In this case, we performed WES (100X estimated depth of sequencing) in both affected siblings according to the guidelines shown in **Supplementary Data Sheet 1**. Information regarding the quality, number of reads, and percentage of mapped reads is presented in **Supplementary Table 1**.

After variant calling, we obtained 312,842 and 301,845 variants for both patients (index case and brother, respectively), of which 119 and 120 were nonsynonymous or located in canonical splice sites with a low population frequency (less than 0.5%), respectively. Of these, 62 were present in both siblings, 57 were missense, four frameshift variants and one stop gain (variants are listed in **Supplementary Table 2**).

**Figure 2:**
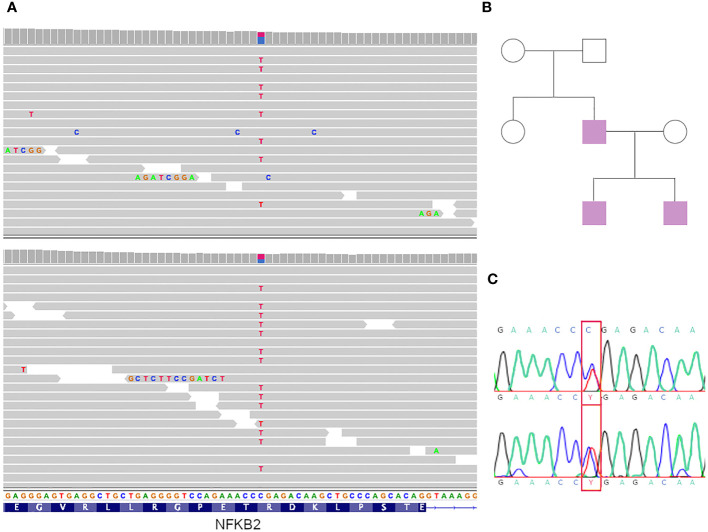
Genomic results and genealogy. **(A)** IGV view of mutations in both siblings. **(B)** Genealogy of the family. In violet affected individuals. **(C)** Sanger sequencing results of the father (patient 3).

The stop-gain variant (which was in a heterozygous state) is in the *NFKB2* gene (NM_001261403:exon21:c.C2557T:p. R853X) and lies towards the end of the protein’s C-terminus (in the 95% of the protein length, **Figure 2A**). This was not reported in the Gnomad cohort, which had a CADD score of 38 and positive phyloP scores (1.016 and 0.365 for phyloP46 and phyloP100, respectively), indicating that it is a potential causative variant. The variant has been reported previously as “Pathogenic” in ClinVar (VCV000065385.32) with a Pathogenic label, with solid evidence of being so, since it has been reported in various affected cases and the gene is known to be associated with common variable immunodeficiency (CVI) with an autosomal dominant inheritance pattern. Most of those patients have a heterogeneous presentation of the disease. Eg. unusual combinations of childhood-onset hypogammaglobulinemia with recurrent infections, autoimmune features, and adrenal insufficiency (2, 30).

Furthermore, even though this variant is not predicted to result in nonsense-mediated decay, it is expected to disrupt the last 48 amino acids of the NFKB2 protein, and experimental studies have shown that this premature translational stop signal affects *NFKB2* function (2).

According to the ACMG guidelines (31), the variant was classified as pathogenic because the following rules apply: PVS1 (nonsense in a gene for which LOF is a known mechanism of disease), PM2 (absence in controls), and PP1 (co-segregation with disease in the family; see below).

### 3.2 Mycosis fungoides as the sole and familial manifestation of common variable immunodeficiency

We analyzed variant segregation in other family members using Sanger sequencing according to the family tree shown in **Figure 2B**. The affected father presented with the variant (**Figure 2C**), which was absent in the unaffected mother.

The immunoglobulin concentration was 319 mg/dL for IgG (reference value 700-1600 mg/dL), 8 mg/dL for IgM (reference value 40-230 mg/dL), 29 mg/dL for IgA (reference value 70-400mg/dL), 450 mg/dL for the brother’s IgG, 20 mg/dL for IgM, and 73 mg/dL for IgA.

The inmunogloblin concentration of the brother was: IgA 73 mg/dL, IgG 450 mg/dL, IgM 20 mg/dL

**Figure 3:**
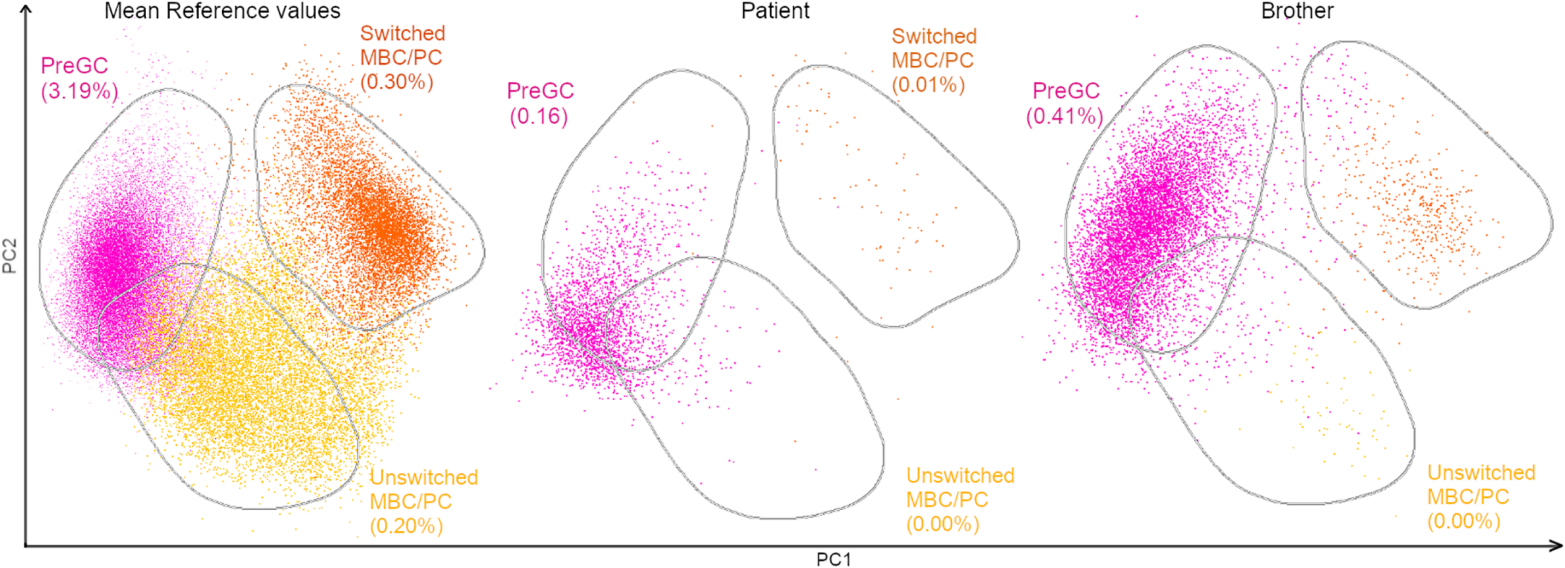
PCA view of flow cytometry PIDOT results of a healthy control, the patient and brother. Multidimensional view (APS view) based on the most discriminating parameters for B-cell subsets. The B-cell subsets were divided into pre-germinal center (PreGC; IgM+IgD+CD27−) unswitched memory B-cells/plasma cells (Unswitched MBC/PC; IgM+IgD+/−CD27+), switched memory MBC/PC (IgM−IgD−CD27+).

Primary immunodeficiency screening and orientation tube (PIDOT) cytometry studies were performed in both siblings and tested positive for decreased immunoglobulin and memory B cell levels (**Figure 3**; **Supplementary Data Sheet 1**). The father did not return for follow-up; therefore, the PIDOT evaluation was unavailable. The affected family members showed no history of infections or autoimmune diseases, and they presented with no other clinical manifestations of CVID except for mycosis fungoides.

Monthly immunoglobulin replacement treatment was initiated for both siblings. The patient is in partial remission and is on the waiting list for a haploidentical transplant with his healthy mother.

## 4 Discussion

Here, we present the case of a family with CVID with an unusual clinical presentation diagnosed by WES. Three family members harbor a mutation in *NFKB2* associated with CVID and they present mycosis fungoides as their only manifestation. To the best of our knowledge, this is the first case series reported in the region.

Patients with CVID have an inherently increased risk of lymphoproliferation, from non-malignant lymphoproliferation to lymphoma. This lymphoproliferative disorder is heterogeneous, with multiple factors including impaired immune surveillance, chronic infection, and genetic predisposition. Compared with the general population of the same age group, patients with CVID had higher rates of lymphoma, leukemia, gastric cancer, and skin cancer. Other known comorbidities of CVID include autoimmune diseases, interstitial lung diseases, bronchiectasis, enteropathies, liver diseases, and granulomatous diseases.

A registry of over 1000 patients with CVID confirmed lymphoma in 4.1% (32). 62% with the lymphoma subtype, 24.4% had a mature B-cell neoplasm, 11.1% had Hodgkin’s lymphoma, and only 4.4% had a mature T-cell neoplasm. A US-based study of 473 patients with CVID found that 8.2% of lymphomas were B-cell subtypes (33). These data are consistent with previous reports, in which the frequency of lymphomas ranges between 2-8% and of them, the T subtype is clearly less frequent; among them, we found no cases of mycosis fungoides (13, 34). However, mycosis fungoides is very infrequent, and familial presentation is extremely rare. Although its etiology is largely unknown, some evidence suggests that genetic and hereditary factors play a role in the pathogenicity of cutaneous lymphomas. To the best of our knowledge, only a few familial MF have been reported (35–39). Unfortunately, there is not much data on the mutational state of NFKB2 in patients with CVID and lymphoma, since the sequencing of *NKFB2* is not part of the diagnosis algorithm of CVID.

Independent studies have reported strong linkage disequilibrium between MF and specific HLA class II allotypes, suggesting significant genetic susceptibility to these diseases (40). A study was conducted based on the Danish Twin Register (a cohort of 42 twins with cutaneous lymphomas (case twins) and their 42 co-twins, 420 age-and sex-matched twin controls (case controls), and 420 co-twin controls over a period of 30 years. The 42 twin pairs comprised 13 monozygotic and 27 dizygotic pairs. Contrary to expectations, neither co-twin was diagnosed with cutaneous lymphoma over the long follow-up period. The first study in a cohort of twins was unable to detect any familial aggregation of these diseases (41).

Father and both sons have a novel stop-gain variant in heterozygosity in the *NFKB2* gene compatible with a CVID phenotype. The variant was classified as pathogenic according to the ClinVar and ACMG rules. Although it is a small family, the disease co-segregates with the variant in the family members studied, since all affected individuals have the variant, whereas the only unaffected relative studied (the sister of the father) does not.

The variant is absent in population frequency databases such as GNOMAD and has pathogenic *in silico* predictors (both conservational and physicochemical). The variant lies towards the end of the gene (at position 853); it has no *in-silico* prediction of activating the nonsense-mediated-decay (NMD) mechanism, but it compromises the last 48 amino acids of the protein, which are key for proper function. Previous studies have shown that NFKB2 protein that was produced from the R853* mutation was able to localize to the nucleus and activate transcription, suggesting that it was functional (no NMD). However, the authors also found that the protein was degraded more rapidly than wild-type NFKB2 protein (42). An additional study showed that R853* mutated protein was able to bind to DNA and activate transcription but was less effective than the wild-type (43).

Activation of *NF-κB2* signaling requires a kinase cascade, resulting in the phosphorylation of p100 (the product of *NF-kB2*) at two conserved C-terminal Serines (Ser866, Ser870) (44) by a kinase called IKKκ. Then, the lysine at position 855 is ubiquitinated, which acts as a signal for the proteasomal processing of p100 to generate p52 (the active form), which associates with RelB and translocates into the nucleus, where this heterodimeric active complex acts as a transcription factor. The premature heterozygous stop codon affects the region of the protein required for posttranslational modifications, specifically phosphorylation and ubiquitination, leading to proteasomal processing. This likely results in reduced (but not completely blocked) protein activation and nuclear translocation as shown in animal models (2). The pathogenesis mechanism in humans with heterozygous NFKB2 mutations results from the haploinsufficiency condition, affecting only the noncanonical NF-κB pathway.

Phenotype refinement was achieved by PIDOT cytometry. It showed a reduced percentage of switched memory B cells (<70% of age-related normal values), a phenotype described in association with LOF variants in *NFKB2*, thus supporting the diagnosis (17).

To the best of our knowledge, hereditary MF as the sole manifestation of CVID is rare. It might have an additional genetic component (not only the NFKB2 variant) detectable in the patient’s genome. Hence, we analyzed other genomic variants present in both siblings with low frequencies and potential pathogenic impacts. We found a heterozygous frameshift variant in the ECM1 gene with a population frequency of 0,000015, that falls within the first 45-48% of the protein (depending on the transcript) and is predicted to activate an NMD mechanism and be damaging. Although variants of this gene are related to a dermatological disorder with generalized thickening of the skin and lipoid proteinosis of Urbach and Wiethe (OMIM:247100), it seems unlikely that this variant has an impact on the phenotype. Both brothers were heterozygous for this autosomal recessive disorder and did not have any elements suggestive of this phenotype. Hence, we did not speculate the role of this variant in ECM1 in this case. However, we do not exclude the possibility that the *NFKB2* variant might act as a first “driver-hit” that might need a second-hit to determine lymphoma development favored by the immunodeficiency.

Since a molecularly defined entity associated with NFKB2 has not been developed yet it is still named as “Immunodeficiency common variable” (see: https://www.omim.org/entry/615577). We propose to depart from the “umbrella diagnosis” of CVID when a specific cause is found (45).

This case highlights the importance of using WES or WGS for proper diagnosis and treatment of hereditary hematological disorders.

## Data availability statement

The original contributions presented in the study are included in the article/**Supplementary Material**, further inquiries can be directed to the corresponding author/s.

## Ethics statement

The studies involving humans were approved by Ethics committee of the Institut Pasteur de Montevideo, Mataojo 2020, Montevideo Uruguay. The studies were conducted in accordance with the local legislation and institutional requirements. The participants provided their written informed consent to participate in this study. Written informed consent was obtained from the individual(s) for the publication of any potentially identifiable images or data included in this article. Informed consent to participate in the study and its publication was obtained from the participants and their families.

## Author contributions

MNS, SG: treated and followed the patient and family. Wrote the manuscript. CS, MB: bioinformatic analysis of whole exome data. Reviewed the manuscript. MBo, SR: followed the patients in the hematological service. AB, JG-L, DL, NT: cytometry analysis. Reviewed the manuscript. VR: Interpretation of variants. Wrote original draft. LS: Bioinformatic analysis, interpretation of variants and funding acquisition. Wrote original draft. All authors contributed to the article and approved the submitted version.

## Supplementary material

SUPPLEMENTARY DATA SHEET 1 Materials and methods. Methodology for sample collection, sample extraction, and sequencing. One section is dedicated to bioinformatics analysis, and the last section contains details on the cytometry analysis (PIDOT).

SUPPLEMENTARY TABLE 1 Quality metrics. Information on the quality, number of reads, and percentage of mapped reads.

SUPPLEMENTARY TABLE 2 Potential candidate variants. Information on the variants found in both siblings.
