# Supplementary material for: Case Report: Mycosis fungoides as an exclusive manifestation of common variable immunodeficiency in a family with a NFKB2 gene mutation
Source: Front Oncol. 2023 Sep 13;13:1248964. doi: 10.3389/fonc.2023.1248964 (PMC10534963; doi:10.3389/fonc.2023.1248964)
Supplement: SUPPLEMENTARY DATA SHEET 1 — Materials and methods. Methodology for sample collection, sample extraction, and sequencing. One section is dedicated to bioinformatics analysis, and the last section contains details on the cytometry analysis (PIDOT). [file DataSheet_1.docx]

**Supplementary data**

**Materials and Methods**

**Sample obtention, sample extraction and sequencing**

Both siblings were referred to our center of hereditary hematological diseases to be included in the genomic pilot study.

Genomic DNA was extracted from 200μl of whole blood using QIAamp DNA Blood Mini kit according to the manufacturer’s protocol. Whole exome sequencing was done with 100X in a HiSeq2500 Illumina sequencing machine. The variant of interest was confirmed in the patients and other family members via Sanger sequencing.

**Bioinformatic analysis**

FastQC [1] was used for the quality of reads, and BWA [2] for read mapping onto the human genome (GRCh37). Variant calling was undertaken using GATK [3] (best practices). Annotation was done with ANNOVAR [4]. Different filters were used in order to detect potentially causative mutations (see below).

In order to filter and prioritize the variants found, we used the following rationale:

1. Heterozygous mutations in coding/splicing regions with a population frequency less than 0.1% in each sibling.
2. Pathogenic variants as reported by the Clinvar (https://www.ncbi.nlm.nih.gov/clinvar) database in each sibling.
3. Variants found in both siblings were further analyzed and prioritized.

**Flow cytometry analysis**

EuroFlow consortium has designed an 8-color antibody panel to orientate in the diagnosis and classification of PID. PIDOT was designed to allow fast and robust detection and enumeration of all main leukocyte and lymphocyte subpopulations in a single 8-color labeling standardized and validated tube. The panel includes antigens for the identification of the different lymphocyte populations (CD45, CD19, CD3, CD16 and CD56), and also markers to further study B (CD27, IgM and IgD) and T (CD4, CD8, TCRγδ, CD27 and CD45RA) cell subpopulations [13, 31, 32]. Blood samples were processed and stained following the EuroFlow standard operating procedures for staining, instrument set-up and calibration, as previously described available at www.EuroFlow.org and the data were acquired on BD FACSCanto II instrument [33]. For data analysis, the Infinicyt software v 2.0.5 (Cytognos SL, Salamanca, Spain) was used. The software allows an automatic analysis option that can be applied to all samples processed, stained and measured according to the standardized EuroFlow protocols. Comparison to a cohort of genetically-defined PID patients and a set of healthy controls generated using normal samples of different age groups (Euroflow PIDOT database) allows performing standardized analysis of this antibody combination [31].

References

[27] Wingett SW, Andrews S. FastQ Screen: A tool for multi-genome mapping and quality control. F1000Res. 2018 Aug 24;7:1338. doi: 10.12688/f1000research.15931.2. PMID: 30254741; PMCID: PMC6124377.

[28] Li H, Durbin R. Fast and accurate short read alignment with Burrows-Wheeler transform. Bioinformatics. 2009 Jul 15;25(14):1754-60. doi: 10.1093/bioinformatics/btp324. Epub 2009 May 18. PMID: 19451168; PMCID: PMC2705234.

[29] McKenna A, Hanna M, Banks E, Sivachenko A, Cibulskis K, Kernytsky A, Garimella K, Altshuler D, Gabriel S, Daly M, DePristo MA. The Genome Analysis Toolkit: a MapReduce framework for analyzing next-generation DNA sequencing data. Genome Res. 2010 Sep;20(9):1297-303. doi: 10.1101/gr.107524.110. Epub 2010 Jul 19. PMID: 20644199; PMCID: PMC2928508.

[30] Wang K, Li M, Hakonarson H. ANNOVAR: functional annotation of genetic variants from high-throughput sequencing data. Nucleic Acids Res. 2010 Sep;38(16):e164. doi: 10.1093/nar/gkq603. Epub 2010 Jul 3. PMID: 20601685; PMCID: PMC2938201.

[13] Ameratunga R, Allan C, Woon ST. Defining Common Variable Immunodeficiency Disorders in 2020. Immunol Allergy Clin North Am. 2020 Aug;40(3):403-420. doi: 10.1016/j.iac.2020.03.001. Epub 2020 Jun 7. PMID: 32654689.

[31] Van der Burg, M., Kalina, T., Perez-Andres, M., Vlkova, M., Lopez-Granados, E., Blanco, E., Bonroy, C., Sousa, A. E., Kienzler, A. K., Wentink, M., Mejstríková, E., Šinkorova, V., Stuchly, J., van Zelm, M. C., Orfao, A., & van Dongen, J. J. M. (2019). The EuroFlow PID Orientation Tube for Flow Cytometric Diagnostic Screening of Primary Immunodeficiencies of the Lymphoid System. Frontiers in immunology, 10, 246. https://doi.org/10.3389/fimmu.2019.00246.

[32] Neirinck, J., Emmaneel, A., Buysse, M., Philippé, J., Van Gassen, S., Saeys, Y., Bossuyt, X., De Buyser, S., van der Burg, M., Pérez-Andrés, M., Orfao, A., van Dongen, J. J. M., Lambrecht, B. N., Kerre, T., Hofmans, M., Haerynck, F., & Bonroy, C. (2022). The Euroflow PID Orientation Tube in the diagnostic workup of primary immunodeficiency: Daily practice performance in a tertiary university hospital. Frontiers in immunology, 13, 937738. https://doi.org/10.3389/fimmu.2022.937738.

[33] Kalina, T., Flores-Montero, J., van der Velden, V. H., Martin-Ayuso, M., Böttcher, S., Ritgen, M., Almeida, J., Lhermitte, L., Asnafi, V., Mendonça, A., de Tute, R., Cullen, M., Sedek, L., Vidriales, M. B., Pérez, J. J., te Marvelde, J. G., Mejstrikova, E., Hrusak, O., Szczepański, T., van Dongen, J. J., … EuroFlow Consortium (EU-FP6, LSHB-CT-2006-018708) (2012). EuroFlow standardization of flow cytometer instrument settings and immunophenotyping protocols. Leukemia, 26(9), 1986–2010. https://doi.org/10.1038/leu.2012.122.
